# Infantile scabies mimicking refractory eczema: a case report and review of the literature

**DOI:** 10.3389/fmed.2026.1919356

**Published:** 2026-08-12

**Authors:** Qingqing Guo, Fangfang Bao, Zhen Li, Tongsheng Chu, Yuan Zhang, Hongqing Tian

**Affiliations:** 1 Dermatology Hospital of Shandong First Medical University, Jinan, China; 2 Shandong Provincial Institute of Dermatology and Venereology, Shandong Academy of Medical Sciences, Jinan, China

**Keywords:** dermoscopy, eczema, infant, palmoplantar pustules, scabies, steroid resistance

## Abstract

Atypical infantile scabies is easily misdiagnosed as common inflammatory dermatoses, leading to therapeutic delay. This 11-month-old female infant presented with two months of generalized pruritus and steroid-refractory palmoplantar pustules, which failed to respond to topical glucocorticoids and calcineurin inhibitors. Epidemiological inquiry revealed familial clustered pruritus and definite occupational scabies exposure in her mother, a ward nurse. Dermoscopy directly identified mites and confirmed the diagnosis. Standardized sulfur ointment treatment for the infant and synchronous eradication therapy for all household members achieved remission. This case highlights that anti-inflammatory-refractory palmoplantar pustules are crucial red flags for atypical infantile scabies. For intractable eczema-like eruptions in infants, targeted contact tracing and dermoscopic evaluation should be prioritized over prolonged empirical anti-inflammatory therapy to enable early and accurate diagnosis.

## Introduction

Infantile scabies remains underrecognized despite rising global incidence (1). Classic adult scabies typically presents with nocturnal pruritus and pathognomonic burrows in flexural skin folds, whereas infants develop diffuse, atypical involvement extending beyond classic sites. Owing to an immature skin barrier function, affected infants frequently exhibit lesions on the palms, soles, and face, with secondary eczematous changes often closely mimicking atopic dermatitis or cutaneous infection (1–5). We report an 11-month-old infant with generalized eruptions and steroid-refractory palmoplantar pustules, initially misdiagnosed as eczema. Epidemiological inquiry identified a definitive occupational exposure link: the patient’s mother, a general ward nurse, had cared for a scabies patient. Dermoscopic visualization of the mite secured the diagnosis. A combined regimen of standardized sulfur ointment for all household contacts and thorough environmental decontamination achieved clinical remission. This case underscores steroid refractoriness and palmoplantar involvement as critical clues for atypical infantile scabies. Routine screening of all household contacts is therefore warranted in children with treatment-refractory pruritic dermatoses.

## Case report

An 11-month-and-18-day-old female infant presented with a 2-month history of generalized pruritic erythematous papules and papulovesicles, notably involving the palms and soles with superimposed pustules. The cutaneous lesions initially emerged on the abdomen and gradually spread to the plantar aspects of both feet. The patient had been repeatedly diagnosed with eczema in external hospitals and had received standardized topical anti-inflammatory treatment, including desonide cream and pimecrolimus cream. However, the lesions showed no improvement and progressively worsened, accompanied by secondary pustule formation on the hands and feet. The infant suffered from severe nocturnal pruritus, manifesting as irritability and severely impaired sleep quality. No systemic symptoms such as fever, cough, or diarrhea were observed throughout the disease’s course.

**Figure 1:**
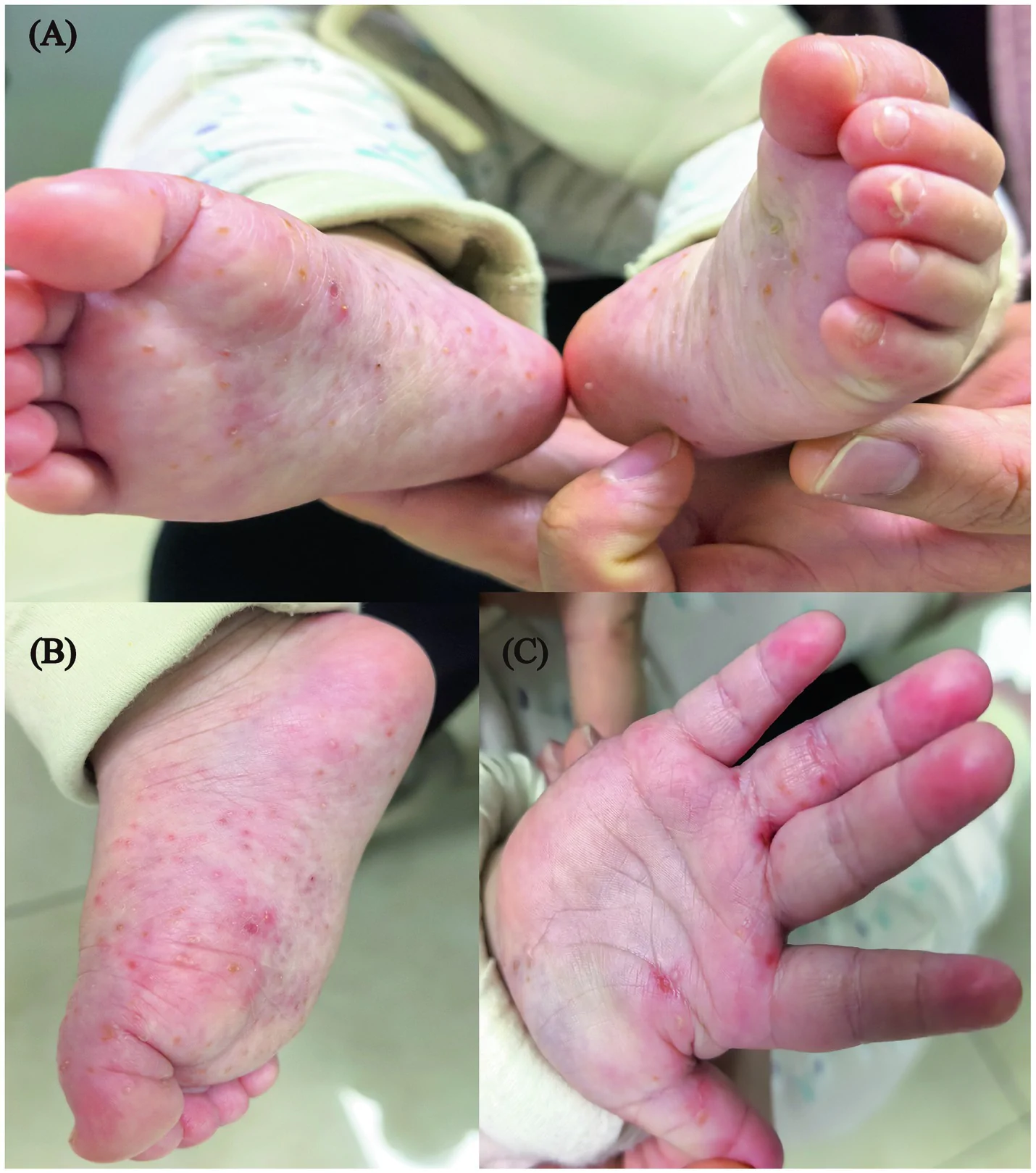
**(A)** Diffuse papulovesicles on both soles; **(B)** scattered pustules on close-up examination; **(C)** characteristic pruritic papules and crusted lesions in the finger webs and over the metacarpophalangeal joints.

Physical examination revealed stable vital signs and unremarkable cardiopulmonary and abdominal findings. Dermatological examination identified scattered erythema, papules, and papulovesicles involving the trunk and extremities, with a predilection for the feet (Figures 1A,B). Multiple pustules were observed on the hands and plantar surfaces. Characteristic pruritic papules and crusted lesions were noted in the finger webs and over the metacarpophalangeal joints (Figure 1C), accompanied by scattered excoriations over the trunk, suggestive of persistent severe pruritus. Given the poor response to conventional anti-eczema agents and the presence of progressive atypical manifestations, we conducted detailed history-taking and familial screening. A detailed history revealed that all household contacts—including the parents and maternal grandparents—had developed unexplained persistent pruritus concurrently, approximately 7 weeks prior to the infant’s diagnosis. Notably, the infant’s mother—a general ward nurse—presented with classic erythematous papules and excoriations in the finger webs and axillae, which are pathognomonic predilection sites for scabies. Further occupational exposure history confirmed contact with a hospitalized scabies patient 9 weeks prior, establishing a definitive epidemiological link.

**Figure 2:**
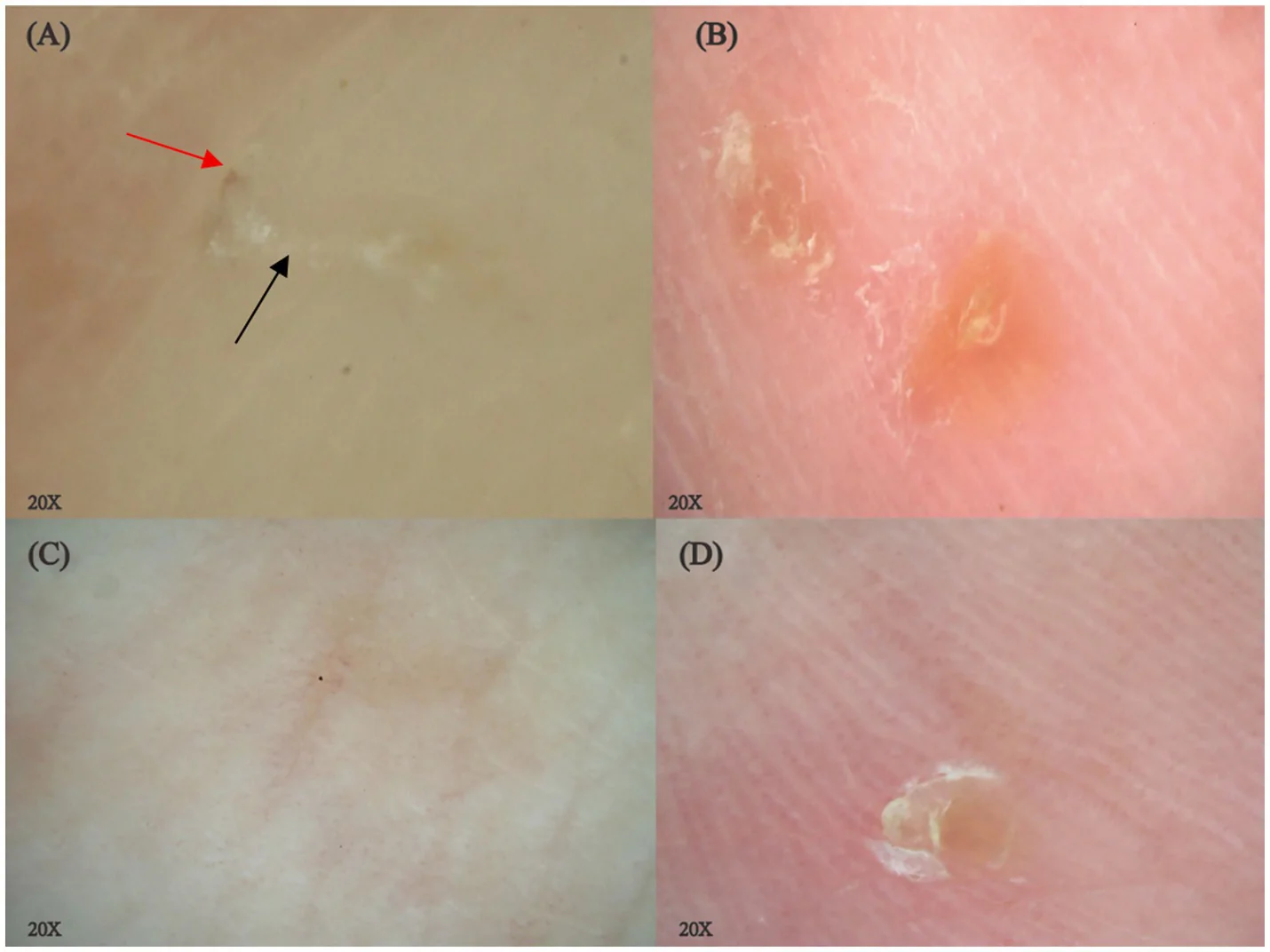
Dermoscopic findings. **(A)** The serpiginous burrow (black arrow) contains a dark triangular structure (red arrow) at its distal end, representing the characteristic “jet-with-contrail” sign of scabies. **(B)** A triangular mite structure is visible around the edge of a pustule. **(C,D)** After 2 weeks of treatment, both images demonstrate diffuse telangiectasias and residual scales, with no burrows or mites (Magnification ×20).

Dermoscopic examination of the foot lesions revealed tortuous burrows, with a dark equilateral triangular structure visible at the distal end of a burrow, a feature commonly referred to as the “jet-with-contrail” sign (Figure 2A), which confirmed the clinical diagnosis of scabies. In addition, a triangular structure was visible around the edge of a pustule (Figure 2B). The infant was treated with 5% sulfur ointment applied to the entire body, including the scalp, for three consecutive days, with bathing and clothing changes suspended during treatment. All personal garments and bed linens were disinfected by boiling. All household family members received synchronous standardized treatment with 10% sulfur ointment applied from the neck down, accompanied by thorough environmental decontamination. A repeated therapeutic cycle was conducted 1 week later to eliminate newly hatched mites.

A 2-week follow-up demonstrated significant clinical improvement. The patient’s pruritus was substantially relieved, and the original erythematous, vesicular, and pustular lesions became dry and crusted with no new eruptions. Dermoscopy confirmed the absence of mites and burrows, revealing only residual scales and telangiectasias (Figures 2C,D). This case highlights that infantile scabies is prone to misdiagnosis as eczema due to its non-specific early manifestations. For intractable generalized pruritic skin lesions unresponsive to conventional anti-eczema treatment, familial epidemiological clues and dermoscopic detection are essential for early and accurate diagnosis, avoiding delayed intervention and recurrent intrafamilial transmission.

## Discussion

Scabies, caused by *Sarcoptes scabiei* var. hominis, remains a critical global health concern, with outbreaks increasingly reported even in high-income regions beyond its traditional low-resource endemic settings (6, 7). The 2021 Global Burden of Disease Study estimates that there are 206.6 million prevalent cases of scabies worldwide (8). The prevalence of this disease varies markedly across regions and populations, with tropical and subtropical zones, especially low- and middle-income countries, bearing the highest disease burden (9). Population-based studies have further shown that scabies prevalence ranges from 0.18 to 76.9% worldwide (10). In addition to its well-known clinical manifestations, scabies can trigger secondary bacterial complications and cause social prejudice against affected individuals (11). Infantile scabies is notoriously difficult to diagnose. Unlike adults, infants often develop lesions on the face, scalp, palms, and soles. This atypical distribution, particularly when presenting as palmoplantar pustules, frequently mimics acropustulosis or eczema, leading to treatment delays. We report a case of steroid-refractory scabies with palmoplantar involvement in an infant, initially misdiagnosed as eczema. The diagnosis was ultimately established through characteristic clinical features, detailed epidemiological inquiry, and confirmatory dermoscopy. This report aims to analyze the clinical features, causes of misdiagnosis, diagnostic key points, and standardized therapeutic strategies to deepen clinicians’ understanding of atypical infantile scabies, reduce diagnostic bias, and improve the accuracy of early diagnosis.

Unlike adult scabies confined to flexural folds (the “circle of Hebra”), infantile disease is notoriously deceptive. A thin stratum corneum allows mites to colonize the scalp and face, as well as the palms and soles, presenting as pustules or eczema. This atypical distribution, compounded by steroid resistance, leads to chronic misdiagnosis (12, 13). The 11-month-old infant presented with palmoplantar pustules, necessitating systematic differential diagnosis. Eczema, palmoplantar pustulosis, infantile acropustulosis, and arthropod bites typically show favorable responses to topical glucocorticoids or calcineurin inhibitors such as pimecrolimus; folliculitis may manifest as pustules but is generally non-pruritic. In contrast, this patient was refractory to both classes of medication and experienced intense pruritus, effectively ruling out the aforementioned differential diagnoses. Against the backdrop of improved public hygiene standards, scabies is increasingly overlooked in routine clinical practice. Epidemiological inquiry revealed clustered pruritus among multiple family members. A critical epidemiological clue emerged with the identification of occupational exposure: the infant’s mother, a general ward nurse, had provided care for a laboratory-confirmed scabies patient 2 months prior. Invasive skin scraping was deemed unsuitable due to the infant’s poor procedural compliance and the family’s explicit refusal of invasive procedures; accordingly, non-invasive dermoscopy was performed. Direct visualization of the scabies mite under dermoscopy confirmed the diagnosis. This case highlights a pragmatic diagnostic approach for infantile scabies: in cases of persistent palmoplantar pustules refractory to topical glucocorticoids, clinicians should prioritize obtaining a detailed history of clustered pruritus among household contacts—particularly when a parent works in healthcare—and pursue prompt dermoscopic evaluation to avoid misdiagnosis as eczema.

The pustular phenotype in infants is highly suggestive of scabies yet is often misdiagnosed as a bacterial infection or severe eczema. Unable to report pruritus, affected infants present with nighttime restlessness and poor sleep, which are commonly attributed to colic, teething, or calcium deficiency, masking the true skin-related cause. Sustained mite infestation and scratching trigger secondary eczematous changes; typical scabies burrows become hidden under scales and crusts, forming lesions that closely mimic eczema. Steroid refractoriness serves as a key clinical alert. Lesions of atopic dermatitis usually improve rapidly with topical steroids or calcineurin inhibitors, whereas scabies eruptions persist because of continuous mite colonization and hypersensitivity to mite antigens. Anti-inflammatory preparations only alleviate superficial symptoms without eliminating mites and their ova. Additionally, glucocorticoids induce local immunosuppression, impair the skin barrier, and facilitate mite multiplication. In the present case, 2 months of ineffective treatment with desonide followed by pimecrolimus ought to have raised clinical suspicion of scabies. Nevertheless, diagnostic bias toward eczema delayed correct identification. We thus recommend a straightforward clinical principle: parasitic assessment should be performed for infants with eczematous rashes unresponsive to topical anti-inflammatory agents prior to treatment intensification.

Atypical infantile scabies is fundamentally a household contagion. Because their contacts are largely domestic, infants acquire mites from cohabitants (6). Familial pruritus is therefore a critical clue, yet clinicians often overlook it, focusing solely on morphology while neglecting contact tracing and occupational risks—a pitfall that contributes to diagnostic delay. Here, pruritus in the parents and maternal grandparents mapped a clear transmission chain. The mother’s occupation as a registered nurse with direct exposure to a scabies patient provided the definitive link, explaining the infant’s refractory rash. Clinically, a family-based evaluation is essential for infants with persistent pruritus. Even without classic burrows or with eczema-like lesions, concurrent caregiver itch should trigger immediate suspicion of scabies.

Dermoscopy effectively overcomes the limitations of traditional diagnostic methods and significantly improves the efficiency of non-invasive early diagnosis (14, 15). Skin scraping microscopy, regarded as the conventional gold standard for scabies diagnosis, has prominent disadvantages in infants (16). The invasive sampling procedure causes pain and poor patient cooperation. Severe inflammatory responses in infantile scabies often destroy superficial burrows, and the low parasite load frequently leads to false-negative results. Additionally, the procedure is time-consuming and may delay treatment. As a rapid, precise, and non-invasive visualization tool, dermoscopy enables direct observation of characteristic scabies structures, including mite burrows, parasite bodies, the classic delta-wing sign, and the gray-line sign without trauma (17). In this case, dermoscopy clearly identified typical mite structures in plantar lesions, achieving immediate and accurate diagnosis. Accordingly, dermoscopy should be recommended as the first-line adjunctive examination for clinically suspected infantile scabies.

Achieving definitive cure and preventing relapse of infantile scabies requires a structured, safety-first approach. For children under 2 years of age, topical permethrin is the most frequently recommended treatment (3, 5, 9). Although oral ivermectin exhibits acceptable safety, its clinical use is limited to complicated infection scenarios (18, 19). Access to preferred scabicides varies significantly across clinical settings, largely due to regional drug supply gaps. For infants, safety should take precedence over efficacy considerations in regimen selection: their immature skin barrier and high transdermal absorption rates heighten the risk of systemic adverse effects (20). Following domestic pediatric consensus guidelines, we selected 5% sulfur ointment as first-line therapy. While malodorous and mildly greasy, the agent poses no risk of systemic percutaneous toxicity. It is far safer than lindane, which is contraindicated in infants due to neurotoxicity, and addresses frequent stock shortages of permethrin in our institution. Isolated treatment of the infant alone cannot block transmission. Scabies has a prolonged incubation period and high rates of subclinical carriage among household contacts; untreated asymptomatic carriers act as persistent reservoirs, driving recurrent cross-infection and pseudo-relapse (apparent recurrence due to reinfection). We implemented an integrated three-part strategy: (1) standardized infant topical therapy, (2) simultaneous treatment of all household contacts, and (3) high-temperature disinfection of all fomites (clothing and bedding). At 2-week follow-up, the infant exhibited near-complete resolution of pruritus and substantial regression of cutaneous lesions. Crucially, dermoscopy confirmed the absence of mites and burrows, revealing only residual scales and telangiectasias. This microscopic evidence validates not only the therapeutic efficacy but also the utility of dermoscopy as a reliable tool for monitoring treatment response in infants.

In summary, infantile scabies manifests diverse and atypical cutaneous features in young infants, frequently leading to misdiagnosis as eczema, impetigo, or allergic dermatitis. Clinicians should avoid a rigid diagnostic bias toward eczema. Scabies should be strongly suspected in infants presenting with palmoplantar and web-space eruptions, steroid-refractory dermatitis, nocturnal restlessness, and a family history of aggregated pruritus. Detailed epidemiological inquiry combined with non-invasive dermoscopy serves as a practical and reliable strategy to improve diagnostic accuracy. Standardized topical treatment, simultaneous anti-scabies intervention for all family members, and rigorous environmental disinfection are essential to terminate intrafamilial transmission, prevent recurrent cross-infection, and improve the overall prognosis of affected infants.

## Conclusion

Steroid-refractory palmoplantar pustules should alert clinicians to infantile scabies. Integrating dermoscopy with targeted history-taking for familial itch and healthcare exposure is crucial to prevent misdiagnosis and avoid unnecessary anti-inflammatory escalation.

## Data Availability

The raw data supporting the conclusions of this article will be made available by the authors, without undue reservation.
